# Extracts

**Published:** 1883-10

**Authors:** 


ATLANTA
### MEDICAL REGISTER.

VOL. III.] OCTOBER, 1883. [No. 1

### Extracts.

### INCREASE OF DOCTORS IN ATLANTA.

We were collecting thought and material for a somewhat lengthy article on this subject. It is urgent that something candid and outspoken should be written and widely published, if by this means this bootless, infatuated professional influx may be checked.

It would be a crime and a cruelty for a real estate agent or any professional man to announce that there is another foot of standing room for a doctor in Atlanta. We were on the eve of performing this duty when we found in the *Medical and Surgical Reporter* the following paper, which so completely covers the ground that the reader has only to imagine Atlanta instead of Philadelphia in view:

SHALL I LOCATE? AND WHERE?—*By George B. H. Swayze, M.D, of Philadelphia, Pa.*—In large cities the floating masses solve annoying pecuniary conundrums by a change of location to other neighborhoods, wherein they are lost from sight of old pursuers. The contingencies of fashion, taste and expedients, whet the wits of a large class who adopt for their moral code "The world owes us a living whether we pay for it or not." This is usually the class in cities who seem to welcome the new physician, to his earlier joy, but his later sorrow. A "new physician" who had been trying city practice for something more than a year, was at last housed in nearly three months by illness. On meeting him one day afterwards, I asked what had been the matter. His answer was a volume compressed into an utterance of outraged feeling: "*Nothing but bad bills! It was nothing but general exhaustion from running after bad bills—and mental worry—they wore me out with faithless promises!*"

Physicians in the country are sometimes prone to think that city doctors do not have to work hard for their money, and the former conclude to remove to the city for an easier field. Unfortunately these often find the field so easy that it becomes necessary to move back again. The writer has lived in his present location eleven years. During that time at least a score of doctors have located in the same neighborhood, tried it a few months or years, and then moved away. Several of these were from the country, men of good practice there, but almost clientless here. In the course of a year or two several wisely returned to their old neighborhoods, where they were known, and doubtless where they were welcomed by appreciating friends—for nowhere does a man feel so lonely and helpless as in the wilderness of a great city of strangers. Because ten physicians succeed in making a living with their offices near each other on a certain street, it is a grave mistake to expect that the prospect promises as much to twenty more because they put out their shingles. A few months ago I counted the signs of more than thirty doctors in the space of seven squares on this street and the immediate cross-streets. One has been removed by disease, one removed himself by suicide, six have sought fatter pastures elsewhere.

Physicians themselves are not always to blame for these mistakes of judgment, especially if they come from the country. Too often they are easy game in the agile hands of adroit real estate agents, who so well know how to praise up the prospects of any neighborhood where they may have a house to let or to sell. To illustrate: Some years ago a well-to-do physician from a large interior town thought he would like to move to this city; found a desirable up-town neighborhood where considerable building was in progress, and consulted a local real estate agent that had houses to rent. The astute agent soon furnished most satisfactory assurances of the wisdom of the doctor's project, and clinched his faith by citing the case of another doctor who had lately located in the same neighborhood, and "succeeded so well that in a year's time he was able to buy a $7,000 house!" Of course the willing doctor rented a house and moved to the city; in six months discovered he had made an unprofitable venture; tried another part of the city for six months more, and then moved back to his old home, a year and two thousand dollars out, besides business lost.

But *does* the city doctor have the easier life? No. One hundred dollars in the country will reach as far as three hundred to six hundred in large cities, according to location. With fees nearly the same among the general rank and file of the profession, it is self-evident that the city doctor must do extra work, must sustain extra wear and tear of body and mind to come out even. The perplexing uncertainties of a city establishment harrass the lives of city physicians by day and by night. Hence, also, that too many city practitioners feel impelled to round out limited receipts by engaging every spare hour needed for rest and recuperation, in the severe toils of authorship in some form. It is the pressure of current expenses rather than the pressure of overwork, that keeps so many pens busy turning out reports, reviews, essays and books; and hence the publication of so much that is speculative, artificial, unreliable and unsatisfying to the mass of practitioners. To practitioners in general in cities, professional experience is not so rich and varied as in country locations. The hospitals, infirmaries and dispensaries in cities, run by a select few to foster reputation in a *specialty*, or to open paths of introduction to general practice, monopolize the great harvest of cases by which beginners are usually enabled to gain prompt foothold and win distinguished success. In cites not the poor only, but many in entirely comfortable circumstances, become inmates in hospitals for treatment in cases of accidents, or special diseases, or they receive "out-door" treatment at their homes through dispensary ministrations; while in country locations all the surgery, obstetrics, eye and ear cases, skin and venereal complaints, and the general scope of medical observation and treatment come fully under the study of the local practitioners, thus immensely expanding their opportunities for securing broad and rich experience and early recognition of professional skill.

A physician's prosperity must be gauged at last not by what he is compelled to spend from year to year, but by what he is enabled to save above the current out-go. Ostentation is not wealth. The necessity for display is an exacting task-maker. As compared with large cities, the advantages of country locations afford the great mass of physiciaus much better and safer fields for experience and practice, for professional and social appreciation, for mental and moral expansion, for lucrative reward and substantial prosperity. Among the unpretentious, honorable peers, the honors of his attainments and office make the worthy physician in the country a head above men—a light to look up to in the community: in the rabble of great cities the physician is too often a head below men—a glimmering luminary lost in the jostle and confusion of lights.

"I don't see how all the doctors *live!*" remarked a moneyed business man to another, within my hearing, on a down-town street. During a few minutes' conference on the sidewalk between these lords of ample means, two physicians had jogged past in their peculiar calash-top vehicles, that here announce the professional calling.

"The half of them go with less than half a living," remarked the one addressed, as they moved down the street together.

Such derisive fling is not made against the respectable physician in country places, where the public know that he has an ample exchequer, and besides the income of a good practice, owns a good farm or grist-mill, or several town properties, acquired by his frugal management of modest yearly profits. In the country such is the general possibility; in the city, the rarest of exceptions.

It was the testimony of a successful, prominent Philadelphia physician, on withdrawing from practice some years ago, that in an experience of a quarter of a century, as his income from practice gradually crept up from six hundred a year to three thousand dollars, the expenses of his advancement and social exactions kept even pace with his earnings, and left him nothing to lay by for future dependence.

And it need not be imagined by physicians in the country that eminent college professors acquire fortunes by practice; many of the greatest and the best have died insolvent. In the accessible calm and relaxation that physicians find in country locations without detriment to pocket and prospects, there is a luxury of comfort unknown to the profession amid the almost pauseless din and hurry of city life. *There* may he always find equivalent substitutes for money, when money there is none; but such is not the case in cities. *There* he may be greeted by refreshing landscape, expansion of scene, quiet for weariness, reflection and sociality; but not so in cities, where all is contracted, walled in, hustle and tussle, with no rest to eye or ear, or nervous system, or head or heart from year to year. Is it any wonder so many long to get away for even a little while to enjoy the restful peace of nature's quiet in God's country?

---

THE ENGLISHMAN'S FIRESIDE.—The following article from Prof. W. M. Williams, a well known scientist of England, published in the *Metal Worker*, is so replete with good sense and valuable facts in home sanitation and comfort, that we make no apology for reproducing it entire. It should be read by every man who has now or expects in the future to found a home:

One of the grossest of our national manifestations of conservative stupidity is our senseless, idolatrous worship of that domestic fetich, "the Englishman's fireside." We sacrifice health, we sacrifice comfort, we begrime our towns, and all they contain with sooty foulness; we expend an amount far exceeding the interest of the national debt, and discount our future prospects of national prosperity, in order that we may do what? Enjoy the favorite recreation of idiots. It is a well known physiological fact that an absolute idiot, with a cranium measuring 16 inches in circumference, will sit and stare at a blazing fire for hours and hours continuously, all the day long, except when feeding, and that this propensity varies with the degree of mental vacuity. Few sights are more melancholy than the contemplation of a party of English fire-worshipers seated in a semi-circle round the family fetich on a keen, frosty day. They huddle together, roast their knees and grill their faces, in order to escape the chilling blast that is brought in from all the chinks of the leaky doors and windows by the very great agent they employ, at so much cost, for the purpose of keeping the cold away. The bigger the fire, the greater the draft; the hotter their faces, the colder their backs; the greater the consumption of coal, the more abundant the crop of chilblains, rheumatism, catarrh and other well deserved miseries. The most ridiculous element of such an exhibition is the complacent self-delusion of the victims. They believe that their idol bestows upon them an amount of comfort unknown to other people, that it affords the most perfect and salubrious ventilation, and, above all, that it is a "cheerful" institution. The "cheerfulness" is, perhaps, the broadest part of the whole caricature, especially when we consider that, according to this theory of the cheerfulness of fire-gazing, the 16-inch idiot must be the most cheerful of all human beings.

The notion that our common fireplaces and chimneys afford an efficient means of ventilation is almost too absurd for serious discussion. Everybody who has thought at all on the subject is aware that in cold weather the exhalations of the skin and lungs, the products of gas-burning, etc., are so much heated when given off that they rise to the upper part of the room (especially if any cold outer air is admitted), and should be removed from there before they cool again and descend. Now, our fireplace openings are just where they ought not to be for ventilation; they are at the lower part of the room, and thus their action consists in creating a current of cold air or "draft" from doors and windows, which cold current at once descends, and then runs along the floor, chilling our toes and provoking chilblains. This cold, fresh air, having done its worst in the way of making us uncomfortable, passes directly up the chimney without doing us any service for purposes of respiration. Our mouths are usually above the level of the chimney opening, and thus we only breathe the vitiated atmosphere which it fails to remove. Not only does the fire-opening fail to purify the air we breathe, but it actually prevents the leakage of the upper part of the windows and doors from assisting in the removal of the upper stratum of vitiated air, for the strong up-draft of the chimney causes these openings to be fully occupied by an inflowing current of cold air, which at once descends, and then proceeds as before stated, to the chimney. If the leakage is insufficient to supply the necessary amount of chilblain-making and bronchitis-producing draft, it has to enter by way of the chimney-pot in the form of occasional spasms of down draft, accompanied by gusts of choking and blackening smoke. It is a fact not generally known that smoky chimneys are especial English institutions—one of the peculiar manifestations of our very superior domestic comfortableness. It is true that in some of our rooms an Arnott's ventilator opens into the upper part of the chimney, but this was intended by Dr. Arnott as an adjunct to his modification of the German stove, and such ventilator can only act efficiently where a stove is used. The pressure required to fairly open it can only be regularly obtained when the chimney is closed below, or its lower opening is limited to that of a stovepipe.

The mention of a German stove has upon an English fire-worshiper a similar effect to the sight of water upon a mad dog. Again and again, when I have spoken of the necessity of reforming our fireplaces, the first reply elicited has been, "What! would you have us use German stoves?" In every case where I have inquired of the exclaimer, "What sort of a thing is a German stove?" the answer has proved that the exclamation was but a manifestation of blind prejudice based upon total ignorance. These people who are so much shocked at the notion of introducing "German stoves" have no idea of the construction of the stoves which deservedly bear this title. Their notion of a German stove is one of those wretched iron boxes of purely English invention, known to iron mongers as "shop stoves." These things get red-hot, their red-hot surface frizzles the dust particles that float in the atmosphere, and perfume the apartment accordingly. This, however disagreeable, is not very mischievous, perhaps the reverse, as many of these dust particles, which are revealed by a sunbeam, are composed of organic matter, which, as Dr. Tyndall argues, may be carriers of infection. If we must inhale such things, it is better that we should breathe them cooked than take them raw. The true cause of the headaches and other mischief which such stoves unquestionably induce is very little understood in this country. It has been falselv attributed to over-drying of the atmosphere, and accordingly evaporating pans and other contrivances have been attached to such stoves, but with little or no advantage. Other explanations are given, but the true one is that iron, when red-hot, is permeable by carbonic oxide. This was proved by the researches of Prof. Graham, who showed that this gas not only can pass through red-hot iron with singular facility, but actually does so whenever there is atmospheric air on one side and carbonic oxide on the other.

For the benefit of my non chemical readers I may explain that when any of our ordinary fuel is burned there are two products of carbon combustion, one the result of complete combustion, the other of semi-combustion—carbonic acid and carbonic oxide. The former, though suffocating when breathed alone or in large proportion, is not otherwise poisonous, and has no disagreeable odor; it is, in fact, rather agreeable in small quantities, being the material of champagne bubbles and of those of other effervescing drinks. Carbonic oxide, the product of semi-combustion, is quite different. Breathed only in small quantities it acts as a direct poison, producing peculiarly oppressive headaches. Besides this, it has a disagreeable odor. It thus resembles many other products of imperfect combustion, such as those which are familiar to everybody who has ever blown out a tallow candle and left the red wick to its own devices. On this account alone any kind of iron stove capable of becoming red-hot should be utterly condemned. If Englishmen did their traveling in North Europe in the winter their self-conceit respecting the comfort of English houses would be cruelly lacerated, and none such would perpetrate the absurdity of applying the name of "German stove" to the iron fire pots that are sold as stoves by English ironmongers.

As the Germans use so great a variety of stoves, it is scarcely correct to apply the title of "German" to any kind of stove unless we limit ovrselves to North Germany. There, and in Sweden, Denmark, Norway and Russia, the construction of stoves becomes a specialty. The Russian stove is perhaps the most instructive to us, as it affords the greatest contrast to our barbarous devices of a hole in the wall into which fuel is shoveled and allowed to expend nine-tenths of its energies in heating the clouds, while only the residual ten per cent. does anything toward warming the room. With the thermometer outside below zero, a house in Moscow or St Petersburg is kept incomparably more warm and comfortable, and is better ventilated (though perhaps not so much ventilated) than a corresponding class of houses in England, where the outside temperature is twenty or thirty degrees higher, and this with the consumption of about one-fourth of the fuel which is required for the production of British bronchitis. This is done by, first of all, sacrificing the idiotic recreation of fire-gazing; then by admitting no air into the chimney but that which is used for the combustion of the fuel; thirdly, by sending as little as possible the heat up the chimney; fourthly, by storing the heat obtained from the fuel in a suitable reservoir, and then allowing it gradually and steadily to radiate into the apartment from a large but not over-heated surface.

The Russian stove by which these conditions are fulfilled is usually an ornamental, often a highly artistic article of furniture, made of fire-resisting porcelain, glazed and otherwise decorated outside. Internally it is divided by thick fire-clay walls into several upright chambers or flues, usually six. Some dry firewood is lighted in a suitable fireplace, and is supplied with only suffiient air to effect combustion, all of which enters below and passes fairly through the fuel. The products of combustion being thus undiluted with unnecessary cold air are very highly heated, and in this state pass up compartment or flue No. 1; they are then deflected, and pass down No. 2; then up No. 3, then down No. 4, then up No. 5, then down No. 6. At the end of this long journey they have given up most of their heat to the twenty-four heat-absorbing surfaces of the fire-clay walls of the six flues. When the interior of the stove is thus sufficiently heated, the fire-door and the communication with the chimney are closed, and the fire is at once extinguished, having now done its day's work; the interior of the stove has bottled up its calorific force, and holds it ready for emission into the apartment. This is effected by the natural properties of the walls of the earthenware reservoir. They are bad conductors and good radiators. The heat slowly passes through to the outside of the stove, is radiated into the apartment from a large and moderately heated surface, which affords a genial and well-diffused temperature throughout. There is no scorching in one little red-hot hole, or corner, or box, and freezing in the other parts of the room. There are no drafts, as the chimney is quite closed when the heat reservoir is supplied. If one of these heat reservoirs is placed in the hall, where it may form a noble ornament and can easily communicate with an underground flue, it warms every part of the house, and enables the Russian to enjoy a luxurious, temperate climate indoors in spite of the Arctic winter outside.

In a house thus warmed and free from drafts or blasts of cold air, ventilation becomes the simplest of problems. Nothing more is required than to provide an inlet and outlet in suitable places and of suitable dimensions, when the difference between the specific gravity of the cold air without and warm air within does all the rest. Nothing is easier to arrange than to cause all the entering air to be warmed on its way by the hall stove, and to regulate the supply which each apartment shall receive from this general or main stream by adjusting its own upper outlet. In our English houses with open chimneys all such systematic, scientific ventilation is impossible, on account of the dominating, interfering, useless and comfort-destroying currents produced by these wasteful air-shafts. I should add that the Russian porcelain reservoirs may be constructed for a heat supply of a few hours or for a whole day, and I need say nothing further in refutation of the common British prejudice which confounds so admirable and truly scientific a contrivance with the iron fire-pot above referred to.

There is another kind of stove which, for the sake of distinction, I may call "Scandinavian," as it is commonly used in Norway, Sweden and Denmark, besides some parts of North Germany. This is a tall, hollow iron pillar of rectangular section, varying from 3 to 6 feet in width, and rising half way to the ceiling, and sometimes higher. A fire is lighted at the lower part, and the products of combustion, in their way upward, meet with horizontal iron plates, which deflect them first to the right, then to the left, and thus compel them to make a long serpentine journey before they reach the chimney. By this means they give off their heat to the large surface of iron plate, and enter the chimney at a comparatively low temperature. The heat is radiated into the apartment from the large metal surface, no part of which approaches red heat. A further economy is commonly effected by placing this iron pillar in the wall separating two rooms, so that one of its faces is in each room. Thus two rooms are heated by one fire. One of these may be the kitchen, and the same fire that prepares the food may be used to warm the dining-room. The fire-worshiper is, of course, deprived of his "cheerful" occupation of staring at the coals, and he also loses his playthings, as neither poker, tongs or coal-scuttle are included in the furniture of an apartment thus heated. People differently constituted consider that an escape from the dust, dirt and clatter of these is a decided advantage.

Of course the stoves of our northern neighbors are costly—maybe very costly when highly ornamental. The stove of a Norwegian "bonder" or peasant proprietor costs nearly half as much as the two-roomed wooden house in which it is erected, but the saving it effects renders it a good investment. It would cost £100 or £200 to fit up an English mansion with suitable porcelain stoves of the Russian pattern, but a saving of £20 a year in fuel would yield a good return as regards mere cost, while the gain in comfort and healthfulness would be so great that, once enjoyed and understood, such outlay would be willingly made by all who could afford it, even if no money saving were effected. Only last week I was discussing this question in a railway carriage where one of my fellow-passengers was an intelligent Holsteiner. He confirmed the heresy by which I had shocked the others, in exulting in the high price of coal and wishing it to continue. He told us that when wood was abundant in his country, fuel was used as barbarously, as wastefully and as inefficiently as it now is here, but that the deforesting of the land and the great cost of fuel forced upon them a radical reform, the result of which is that they now have their houses better warmed and at a less cost than when fuel was obtainable at one-fourth of its present cost. Such will be the case with us also if we can but maintain the present coal famine during one or two more winters, especially if we should have the further advantage of some very severe weather in the meantime. Hence the cruel wishes above expressed. The coal famine would scarcely be necessary if we had Russian winters, for in such case our houses, instead of being as they are, merely the most uncomfortable in North Europe, would be quite uninhabitable. With our mild winters we require the utmost severity of fuel prices to civilize our warming and ventilating devices.

---

Anteflexion of the Uterus with Stenosis of the Cervical Canal and Dysmenorrhœa, Treated by Rapid Dilatation. Flatulent Distention of the Stomach.—*William Goodell, M.D., in Medical and Surgical Reporter.*—While our first patient is being etherized, I shall make a few remarks in regard to her condition. She is 25 years old. Puberty began at the age of fourteen, and she has had ever since obstinate and severe dysmenorrhœa. She married two years ago, and has since gone from bad to worse. She comes to us now not so much for the pain during menstruation, but for pain in coition. We have a technical term for painful coition; it is dyspareunia. In other words, she has dyspareunia.

Let us try to trace this matter up; but as I have not yet examined the woman, I may not be entirely correct in my conclusions. When an unmarried woman, or one who is married but has never borne children, complains of dysmenorrhœa, there will be found in the great majority of cases an anteflexion. This is the natural condition of the womb. Retroflexion and retroversion are not normal, but are usually produced by lack of involution after labor. The womb being too heavy topples over backward, and there is produced according to the plasticity of the womb, a retroflexion or a retroversion. If it is plastic and easily bent, there is a retroflexion, but if the uterine tissue is firm, and the ligaments somewhat relaxed, there is a retroversion. This girl, the history tells us, has had dysmenorrhœa since puberty; that she married at the age of 23, and has gone from bad to worse. As I have often told you, marriage without children is poison to the woman. The fact that she has painful menstruation shows that there are two conditions, or one of the two conditions present. There is either an exaggerated anteflexion or a stenosis of the cervical canal, or both. If the bend in the womb is great, no fluid can escape. The blood collects in the cavity of the womb, gradually distending it and straightening the canal, when the menstrual fluid escapes with a gush. The stenosis may be due to the bent condition of the canal. I am in the habit of illustrating it in this way: If when you are watering flowers with a hose, a mischievous boy bends the pipe, a stenosis by angulation will be produced, and the water will stop flowing. In typical cases of dysmenorrhœa from this cause, the menstrual fluid that has collected escapes with a gush; but often when you question the patient you will find that this has not been recognized. They will, however, tell you that the pain is suddenly diminished; that it goes on increasing until it reaches an acme, when there is a lull. It is often difficult to decide whether a woman has stenosis or angulation. The sound may readily pass through the cervical canal, but at the monthly periods the mucous membrane becomes congested and thickened, thus occluding the canal. In married women who do not bear children there is superadded to this monthly congestion, the engorgements from coition. Accumulation of the fluid within the uterus at the menstrual periods leads to hypertrophy of the organ, so that we have in such cases not only the monthly congestions but also the engorgements from coition acting upon this womb already hypertrophied and engorged. There is thus produced a sub-acute form of endometritis. The ovaries also become turgid and tender. When a woman tries to avoid having children by the use of preventive measures, I have seen produced the most exquisite sensitiveness of the cervix. Ordinarily, the cervix is not at all sensitive. A red hot iron can be applied, and the woman will notice only the smell of burnt flesh; caustic may be applied, or it may be stuck with a knife, and the woman not complain. Under certain circumstances, the cervix will become exceedingly tender; and I have found nothing produce it so frequently as the use of preventive measures, especially the one most commonly practiced, that of withdrawal. I have seen a number of such cases. In the first one, the cervix was so tender that I could not touch it, and the moment I attempted to introduce the sound, the woman shrieked. I could not understand it. In telling me her history, she told me that she was using preventive measures, and told me the plan. In this patient I subdued the sensitiveness by the use of vaginal suppositories containing

℞. Morphiæ sulph.,

Ex. Belladonnæ, aa gr. 1.

The next case was a physician's wife. I found the cervix exceedingly tender, but by the exercise of great care I was able to introduce the sound. I then told her directly that she was trying to avoid having children, and told her the plan adopted. She admitted that it was so. I told her that if she wanted to get rid of the trouble that she must avoid the practice. I have seen several other instances, in all of which the women were practicing measures to avoid conception. In withdrawal, the act is not completed on the part of the woman, and the womb is left in a state of terrible congestion. In the act of coition the parts are gorged with blood, but when the fruition of the act is accomplished there is great relaxation. Indeed, this is so great that there is an old Latin adage which says, "After coition, melancholy comes." But when the act is not completed, the womb is left in a state of turgidity for a long time.

This woman is anxious to have children, and it is evident that her condition does not result from any evil practice. She is also naturally desirous that coition should take place in a normal manner with full enjoyment on her part.

Anteflexion *per se* needs no treatment, but if it causes dysmenorrhœa, it calls for treatment unless the dysmenorrhœa be due to nervous causes or irritability of the womb. Irritability of the womb was first described and insisted upon by Dr. Hugh L. Hodge, formerly Professor of Ostetrics in this University. He was so in the habit of dwelling on it that it got to be a sort of a cant phrase among the students, and on the walls of the old University building on Ninth street could be seen many a pencil engraving of Hodge on an irritable uterus. There is a great deal of truth in his teachings in regard to irritable uterus.

I shall now examine the patient. In find that there is an anteflexion, for I can readily get the womb between my two hands, making pressure from above. I pass the sound by pressing it well back against the perineum. It gives a measurement of three inches.

In treating such a case, dilatation is the method, not the cutting, or, as it is called, the bloody operation. This latter consists in slitting up the posterior lip of the cervix to the vaginal junction and passing in a knife and cutting the little spur of tissue within the canal. This is not as successful as dilatation and far more dangerous. Many lives have been sacrificed by it. I have notes of

140 cases in which I employed dilatation, and I have performed the operation in a number of other cases of which I have no notes, and in only a few instances has there been any peritonitis, and in these, it has been rather a slight metritis, with a little implication of the periuterine peritoneum. I have never had any alarming symptoms.

I next pass to the speculum and catching the cervex with a tenaculum, introduce Ellinger's dilator with the curve downwards. This readily enters the cavity. If it will not pass in the first time, pass it as far as it will go and then dilate. You can probably then pass it in to the womb. If nct push it a little further and again dilate. There should always be a shoulder on the instrument two inches from the tip, in order to prevent it from entering too far and hitting the fundus. There should be at least half an inch between the ends of the dilator and the fundus of the womb, for if they were in contact with the womb they would, when opened, be very likely to tear the uterine tissue and lead to serious results. The body of the womb is very vulnerable, while the cervix is not, except to cutting injuries. The dilator passes in so readily that you would say that there is no stenosis, but the stenosis results from swelling of the mucous membrane at the menstrual period. In dilating I open the blades gradually, as I do not wish to exert too much force at once. I have torn the cervix while dilating. One instance was in a married woman who had dysmenorrhœa. When a married wsman who has born children has painful menstruation, it is, as a rule, due to one of two things. The womb is either badly retroflexed, or else she has been treated with nitrate of silver. Twenty years ago every woman, no matter what was the ailment of the womb, who received local treatment, had nitrate of silver applied to the cervix. Many of the older phyeicians are still using it, but it is an exceedingly bad practice. It is sometimes useful, but only in exceptional cases. It causes contraction and hardening of the tissue. In the lady alluded to, nitrate of silver had been applied to the cervix, until the parts had lost all elasticity. In dilating I tore the neck of the womb. The tear did not amount to much, but there was some hemorrhage. This I controlled by Monsel's solution, and the application of a tampon. Once, in a virgin, I tore the cervix, but no bad results accrued. The only thing was that a physician who saw that tear might insist that she had borne a child.

This is an operation which I can confidently recommend. I sometimes have to repeat the dilation, but the woman is so much benefited by the first operation that she readily consents to its repetition. This method of dilating the cervix is not worth anything for the removal of growths within the cavity, for the stretching is not sufficiently great, and the parts come together too quickly.

Having opened the blades to their fullest extent, I have the ether removed and allow the instrument to remain until she begins to squirm. Before beginng the operation I introduce an opium suppository into the rectum. By the time that the effects of the ether have disappeared the opium will have been absorbed. She will be put to bed, and in two hours a second suppository may be required. Two are usually sufficient.

---

A Point in the Progress of Therapeutics.—Whatever encouragement there may be to the medical profession in contemplating the steady decrease of the general death rate of late years, the practical mastery attained over the great pestilential diseases of bygone times, and the increasing recognition accorded by the public to the earnestness and intelligent character of our efforts to prolong the average duration of human life, there are other considerations upon which, although they may be less striking, physicians may found a just sense of satisfaction with the practical working of their craft. Not the least of these considerations is the reflection that the practice of medicine tends constantly toward the greatest possible amelioration of the sufferings, and even the minor discomforts, that to some extent are still inseparable from the state of sickness.

It is within the memory of those now in active life, not only that the course of medication thought necessary in their childhood was annoying, disturbing, and often even prostrating, but that the restrictions put upon them in respect to their eating and drinking, to say nothing of the other features of their daily life, were in many instances harder to bear than the derangements incident to the disease. The worst of it was, that much of the harrowing enginery of the past was in no wise inimical to the continued progress of the morbid condition from bad to worse. Indeed, the man would be bold who should affirm that it was not in many cases directly contributory to such progress. It is not pleasant to reflect that in old times—and not so very old—men were made miserable with the result of increasing their chances of speedy burial, and that this grim fact was the most prominent fruit of centuries of devotion to the study of the laws of health and disease. We may comfort ourselves with the thought that our fathers were led into these errors by faulty methods that we have now abjured—mainly by enslaving their observation at the behest of authority.

Among the most gratifying features of the modern way of managing disease is the consonance of attempts to make patients comfortable with the real agencies at work for their restoration to health. A contributor to our last issue calls attention to one of the more important of these attempts—the free use of water in the dietary of young children. The arguments presented by Dr. Remsen, and the facts that have led him to bring them forward, are such as to command the earnest attention of our readers. There are few restrictions, whether in health or in disease, more vexatious than a limitation set upon the amount of water one is at liberty to drink—amounting often, in the case of infants, to a total prohibition. Since there seems good reason to suppose that this enforced deprivation is as damaging as it is annoying, and since there can be no question that those morbid conditions are very rare in which the unrestricted use of water is injurious, there can be no doubt that the hint given by our contributor will be borne in mind by many a practitioner who might otherwise have overlooked the matter for years.—*New York Medical Journal.*

Cactus Grandiflorus in Sexual Exhaustion.—Dr. Pitzer (*American Med. Journal*) says that while other remedies are required to effect a permanent cure, nothing will give more speedy relief in this condition than cactus grandiflorus. It immediately strengthens the cardiac plexus of the sympathetic and improves cardiac nutrition of the heart. The pulse becomes regular. The expression is hopeful, and past sufferings seem to have been only dreams. It may be said that these symptoms are all secondary, and that cures cannot result from drugs prescribed at these. This is true, but no drug, no matter how effective in healing the original disease, can possibly effect its purpose so certainly and so speedily while the patient is laboring under the terrible nervous symptoms above narrated, which so quickly pass away under the influence of cactus. The primary disease has sometimes, to a great extent, disappeared, but continued long enough to excite the cardiac neuroses referred to. This secondary lesion has existed so long that it does not readily pass away, though the original disease be gone. Here the cactus is not only indirectly curative, but it cures in fact. In all these cases:

℞—Tinct. cactus grand......................ʒj

Aquæ.................................℥iv

M. Sig.—One teaspoonful four times a day. In some cases it is combined with pulsatilla, in others macrotys.—*Gaillard's Medical Journal.*

---

A Peculiar Case.—The *Med. Record*, June 23, 1883, tells us that Professor Porro recently performed an operation for no other purpose than to determine the sex. The individual requiring such unique interference was a pseudo-hermaphrodite, aged twenty-two, had been brought up as a female, although all her (his) tasks had been masculine. The face and chest were like those of a man, although the breasts were considerably developed. The pelvis and lower limbs were like those of a woman. The mons veneris was slightly developed. There was a vulva, with a well-developed clitoris, labia and a short vagina; no penis, no prostate, and no uterus. At the summit of what corresponded with the labia majora, near the inguinal region, two round bodies could be felt, which on pressure gave pain. The question was, were they ovaries or testicles. Professor Porro cut down on the right side, and found that the body was a testicle with epididymis and spermatic cord attached. The wound was closed up, the patient pronounced a man, and sent forth enchanted with his new status in society.

---

The Therapeutic Value of Corn Silk.—Gaillard's *Medical Journal* condenses from *Progres Medicale* an interesting article on the subject of the therapeutic properties of corn silk. The observations upon which the article is based are by Dr. Landrieux. The deductions are : (1) That corn silk is not only a valuable alterant of the urinary secretions, but possesses also an incontestible diuretic value; (2) that the diuretic action of the drug is more or less permanent, the increase in the amount of urine voided continuing three or four days after the administration of the last dose; (3) the results of the diuresis thus produced are observable not only in the urinary apparatus, but in effects produced upon the general circulation.

---

Oleate of Copper for Freckles.—Dr. J. V. Shoemaker, of Philadelphia, states as the result of a somewhat extensive experience, that the careful application of a small piece of the ointment of the oleate of copper at night upon retiring, will usually remove freckles. The oleate of copper ointment is prepared by dissolving one drachm of the salt in a sufficient quantity of oleo-palmitic acid to make a soft ointment.

---

Dr. Squibb has substituted for the ordinary blue and red litmus paper a single color, namely, purple. This purple litmus paper turns red with acids, blue with alkalies. It is claimed to be much more delicate and convenient.

